# A Cluster Randomized Controlled Trial Comparing the Efficacy of Pre‐School Language Interventions—Building Early Sentences Therapy and an Adapted Derbyshire Language Scheme

**DOI:** 10.1111/1460-6984.70036

**Published:** 2025-04-26

**Authors:** Cristina McKean, Christine Jack, Sean Pert, Carolyn Letts, Helen Stringer, Mark Masidlover, Anastasia Trebacz, Robert Rush, Emily Armstrong, Kate Conn, Jenny Sandham, Elaine Ashton, Naomi Rose

**Affiliations:** ^1^ Speech and Language Sciences Newcastle University Newcastle upon Tyne UK; ^2^ Department of Education University of Oxford Oxford UK; ^3^ Division of Psychology Communication and Human Neuroscience University of Manchester Manchester UK; ^4^ Derbyshire Language Scheme Nottingham UK; ^5^ School of Medicine Sunderland University Sunderland UK; ^6^ Finn Coral Statistical Consultancy Edinburgh UK

**Keywords:** intervention, language, preschool, randomized controlled trial, usage‐based

## Abstract

**Background:**

Children's language abilities set the stage for their education, psychosocial development and life chances across the life course.

**Aims:**

To compare the efficacy of two preschool language interventions delivered with low dosages in early years settings (EYS): Building Early Sentences Therapy (BEST) and an Adapted Derbyshire Language Scheme (A‐DLS). The former is informed by usage‐based linguistic theory, the latter by typical language developmental patterns.

**Methods:**

We conducted a pre‐registered cluster randomized controlled trial in 20 EYS randomized to receive BEST or A‐DLS. Children aged 3;05–4;05, who were monolingual, with comprehension and/or production scores ≤ 16th centile (New Reynell Developmental Language Scales—NRDLS) and no sensorineural hearing impairment, severe visual impairment or learning disability were eligible. A total of 102 children received the intervention. Speech and language therapists delivered interventions with high fidelity in 15‐min group sessions twice weekly for 8 weeks. Baseline (T1), outcome (T2), and follow‐up (T3) measures were completed blind to the intervention arm. Outcomes were NRDLS comprehension and production standard scores (SS), measures of language structures targeted in the interventions and communicative participation (FOCUS‐34).

**Results:**

Both interventions were associated with significant change from T1 to T2 and from T1 to T3 in all outcomes. There were no differences between interventions in gains in NRDLS comprehension SS at T2 or T3. BEST produced greater gains in NRDLS production SS between T1–T2 (*d* = 0.40) and T1–T3 (*d* = 0.55) and in BEST‐targeted sentences (*d* = 0.77). Children receiving BEST made significantly more progress after intervention (T2–T3) in both comprehension and production. Both interventions were associated with large, clinically significant changes in communicative participation as measured by teacher reports (FOCUS‐34).

**Conclusions:**

A low‐dosage intervention can produce language gains with moderate to large effects. The accelerated progress after the BEST intervention underscores the significant potential of interventions designed with reference to usage‐based theory, which precisely manipulates language exposure to promote the specific cognitive mechanisms hypothesized to promote language learning.

**WHAT THIS PAPER ADDS:**

*What is already known on the subject*

- Early language development sets the stage for children's educational and psychosocial development and their life chances into adulthood. Early language interventions can be effective; however, there is a need to develop and evaluate early interventions which bring large effects and which can be delivered within the constrained resources of early years provision. Usage‐based linguistics have not been explicitly applied to the design of early language interventions. There is evidence that the Derbyshire Language Scheme (DLS) promotes positive outcomes in comprehension abilities and BEST in production.

*What this paper adds to the existing knowledge*

- Findings from a cluster‐randomized controlled trial demonstrate that BEST, an 8‐week, 15‐min, small‐group intervention, delivered twice weekly can produce moderate to high effects in expressive language outcomes for 3–4‐year‐old children with low language. A‐DLS and BEST bring similar gains in comprehension standard scores but BEST leads to larger and more sustained progress in expression. Faster progress after intervention for BEST supports the hypothesis that it promotes the development of abstract representations of predicate‐argument structures, supporting generalization and accelerating language learning.

*What are the potential or actual clinical implications of this work?*

- BEST, a low‐dosage, manualized intervention delivered with high fidelity can be effective for children from a range of socio‐economic backgrounds bringing moderate to high effects. Effective and efficient intervention can be delivered through the precise manipulation of active ingredients within intervention sessions (in this case, the cognitive mechanisms hypothesized to promote language learning and abstract knowledge in usage‐based theory).

## 1 Introduction

Children's language abilities set the stage for their education, psychosocial development and life chances across the life course. Children with low language at school entry have substantially increased risks of difficulties with literacy, educational attainment, mental health, quality of life, social inclusion and employment (Hulme et al. 2015; Law et al. 2009; Le et al. 2021; Schoon et al. 2009, Schoon et al. 2010; Tomblin 2014; Törnqvist et al. 2009). The social gradient in language abilities due to the effects of poverty and wider family socio‐economic circumstances has long been recognized (Reilly and McKean 2023; Reilly et al. 2014). It has been brought into yet sharper focus by the COVID pandemic with effects of social restrictions disproportionately affecting the language of socially disadvantaged children (Tracey et al. 2022) bringing extreme pressures to bear on a depleted early years workforce (Axford et al. 2015; Eadie et al. 2021; Early Years Alliance 2021).

Several preschool language interventions have proven efficacy (Bleses et al. 2018; Frizelle, Mullane, et al. 2021; Law and Charlton 2022; Law et al. 2017; West et al. 2024) with small to moderate effect sizes (ESs). However many do not fit available resources or service delivery models, making implementation difficult and inequitable (Greenwood et al. 2020; McKean and Reilly 2023; Snowling et al. 2022). Children's services need access to a range of interventions with proven efficacy to choose approaches that best fit the needs of the populations they serve, align with the constraints of service provision, and bring lasting benefits to children.

This study compares the efficacy of two preschool language interventions delivered with low dosages in early years settings (EYS) (Frizelle et al. 2021b). Head‐to‐head comparisons of interventions are rare but provide valuable practical and theoretical insights (Frizelle et al. 2021a, 2021b). Comparisons of effective interventions enable informed choices regarding which works best for a given child, context, family preference or outcome. Comparing interventions delivered with the same dosage, delivery context, level of treatment fidelity and similar resources, tests whether it is the specific learning mechanisms/active ingredients exploited by the interventions which promote change or simply ‘therapy general’ effects (Frizelle and McKean 2022). This study compares the efficacy of two interventions: Building Early Sentences Therapy (BEST) (McKean et al. 2013) and an adaptation of the Derbyshire Language Scheme (DLS) (Knowles and Masidlover 1982). Both interventions aim to develop children's use and understanding of simple sentences with 2, 3 and 4 clauses. In a quasi‐experimental pilot study, Trebacz et al. (2024) found that BEST produced greater standard scores (SSs) gains in expressive language than a treatment‐as‐usual control but not comprehension. In an RCT, Broomfield and Dodd (2011) demonstrated that DLS was associated with improvements in comprehension but not production when compared to a wait‐list control. Comparison between two active interventions is clearly a more stringent research design than comparisons with waitlist controls or treatment as usual (TAU), bringing smaller ESs but also greater confidence that any differences found can be attributable to the intervention.

BEST is based on usage‐based linguistic theory (Tomasello 2000) and systematically manipulates the nature and quantity of the language a child hears to promote the development of abstract representations of predicate‐argument structures (PASs) and hence enable the flexible use of a range of sentence structures (McKean et al. 2013). By promoting abstract representations of PAS, the authors hypothesize that BEST can accelerate future language learning (Langacker 2000) through the memory and processing advantages which abstract knowledge affords (for detailed theoretical background, see McKean et al. 2013; Trebacz et al. 2024).

Usage‐based or constructivist theories posit that the adult end state of language acquisition consists of an inventory of constructions linked to the pragmatic and semantic functions they communicate, rather than a set of grammatical ‘rules’ (Croft and Cruse 2004). These constructions vary along a continuum of abstractness with respect to the lexical items which can be placed into them; constructions range from the highly concrete and inflexible (e.g. ‘How do you do?’) to the highly abstract, and flexible (e.g., NOUN1 + VERB + NOUN2 − meaning NOUN1 acts on NOUN2 and NOUN2 is affected), with other constructions falling somewhere in between (e.g., X wouldn't Y let alone Z).

Tomasello described a usage‐based, constructivist account of language acquisition from words to adult ‘grammar’ (Tomasello 2000, 2003). Once multiword utterances begin to be used, language constructions are posited to proceed through five phases as follows: (1) frozen phrases; (2) lexically specific constructions; (3) abstract constructions; (4) paradigmatic categories; and (5) retreat from over‐generalization. BEST aims to support pre‐school children to develop their knowledge and representations of 2‐, 3‐ and 4‐clause sentences and move through the first three stages from frozen phrases to item‐based constructions to abstract representations.

Tomasello's account also describes the cognitive mechanisms brought to bear on the learning process which allow children to move from one stage to the next. BEST manipulates the language input and learning context to support the child's use of these cognitive mechanisms. BEST exaggerates the qualities of the input and provides additional cues to make these cognitive mechanisms more available to children with and at risk of language difficulties. BEST is designed to exaggerate the features which promote intention reading, cultural learning, categorization, schematization and analogy, and promote mapping and retention, thus supporting the development of abstract representations. Active ingredients manipulated in BEST include the use of joint action routines with turn‐taking; modelling of actions with toys to support mapping of meaning and of argument structure roles; massed modelling of sentences with a systematic variation of nouns around verbs; alignment of sentence models with the same PASs but differing verbs; signing both content words, to support mapping, and morphology to draw attention to the morphological frame. The stages of multiword development, relevant cognitive mechanisms and relevant active ingredients used in BEST are summarized in Appendix 1 in the Supporting Information section.

The DLS, widely used in the UK (Knowles and Masidlover 1982; Roulstone et al. 2012), is based on research describing the stages of typical language development (Bloom and Lahey 1978; Brown 1976). DLS provides a structured syllabus of activities that is individualized to the child's language level, aiming to improve both comprehension and expression. Children are supported to understand and use sentences of increasing length and complexity through play‐based activities where the number of ‘information‐carrying words’ a child is asked to understand or use, gradually increases, beginning with their current ‘word level’ and building incrementally. A structured language teaching approach is taken where a game is created to practice understanding and elicit expressively the specific language structure being targeted. The language used should look and sound as natural as possible, and the games are created so that there is a real pragmatic motivation for the child to engage in the activities and the communication which is central to the game. Children progress through ‘word levels’ (WL) indicated by the number of information‐carrying words in the target sentences (e.g., 1WL − objects; actions; 2WL − object + place; object action; 3WL person + action + object; object + place (including adjective); 4WL − person + action + place (including adjective)). Joint action routines are created within structured activities which constrain the language used and the language required to be understood. Role reversal is used such that children take turns with the adults and other children to follow instructions and take the role of teacher to provide instructions or descriptions. A range of prompting and support if children make errors and/or to promote progression are detailed in the programme, including ‘bridging’ where tasks are made easier by manipulating the context to reduce the WL, cloze procedures, recasting and error correction.

Based on previous research, we hypothesize that BEST and an adapted DLS (A‐DLS) will be associated with positive change, with greater gains in children's production from BEST and in comprehension from DLS. Based on underpinning theory and due to the hypothesized promotion of abstract representations allowing knowledge to be generalized, we hypothesize that BEST will bring greater benefits in non‐targeted structures, and accelerated progress after the intervention.

### 1.1 Research questions

- Which intervention brings greater gains in language production and comprehension?
- Do interventions differ in the degree to which benefits transfer to non‐targeted language structures and/or communicative participation?
- Do interventions differ in the degree to which language abilities continue to improve post‐intervention?

## 2 Methods

This pre‐registered cluster randomized controlled trial took place in three local authorities (LAs) in England between January 2020 and June 2022 (ISRCTN10974028) (McKean et al 2020) and is reported with reference to CONSORT guidance (Campbell et al. 2012) (see Appendix 2 in the Supporting Information section). A total of 20 EYS were allocated to receive either BEST or A‐DLS in two waves to avoid contamination within an EYS and enable group delivery. A simple power calculation using Cohen's power tables (Cohen 1988) was completed. At 80% power, two‐tailed *α* = 0.05 and an estimated ES of *d* = 0.5 (derived from Hagen et al. (2017) the most similar recent trial), the sample required was 65 children in each arm. A target of 72 in each treatment arm was set to allow for the 6% attrition found in a study pilot which was conducted in three settings in areas of social disadvantage through student dissertations at Newcastle University. The aims of the pilot were to determine the most appropriate outcome measures, levels of need, recruitment and retention rates and acceptability of study processes to parents/caregivers and settings. As the study coincided with the COVID pandemic, some modifications were needed, details of which are provided in the registered protocol (see ISRCTN10974028). The most significant change was the removal of a TAU arm. The team and participating schools felt it was unethical to assess children's language without offering additional support and intervention at this time when children's language, communication and social–emotional well‐being were at such high levels of risk. The sample in each treatment arm was slightly lower than originally planned due to the loss of time when the UK was in full lockdown, reducing the number of waves of active data collection from three to two.

Treatment was delivered by research assistants (RAs), who were qualified speech and language therapists (SLTs), to 102 preschool children twice a week for 8 weeks: 10 EYS in each of two waves. Newcastle University's ethics committee gave ethical approval. Parents/carers, headteachers and EYS staff provided fully informed consent.

### 2.1 Recruitment of EYS

LA early years advisers and/or SLT managers were approached to act as gatekeepers and asked to invite EYS they identified as having high levels of need to an information event (i.e., where there were known to be high proportions of children not meeting the UK statutory assessment Early Years Foundation Stage Profile (EYFSP) expected levels for Communication and Language; and/or had high levels of referrals to and requests for advice from SLT services). A total of 36 EYS completed an expression of interest to be considered for the study. EYS with the proportion of bilingual children higher than the average in England (20%) were excluded (*n* = 8) and invited to participate in a study for non‐English delivery of BEST. A total of 24 of the remaining 28 were chosen at random for participation by a statistician external to the study as this was the maximum number of settings where intervention could be delivered within the capacity of the RA team. After the pause in the study due to the COVID pandemic, 7 EYS withdrew and a further four were invited to join. The resulting 21 EYS were randomized to receive BEST or A‐DLS.

### 2.2 Randomization

Randomization of EYS to one of two intervention arms was conducted by a statistician not involved with the study. Ten settings were randomized for Wave 1 participation and 11 for Wave 2. To enable the delivery of the intervention to the maximum number of children, nurseries were grouped into geographical clusters. Randomization applied the minimization method (Altman and Bland 2005), stratifying by geographic cluster and social disadvantage (high/low). High and low social disadvantage was assigned using a median split in the proportion of pupils in each school eligible for Pupil Premium: a UK government school subsidy provided for children meeting criteria of social disadvantage (www.gov.uk/government/publications/pupil‐premium/pupil‐premium). Minimization aims to balance these factors across the treatment arms (Table 1). This technique randomly chooses participants from the available pool (in this case EYS) and then assigns them to intervention arms in turn in a manner which best maintains the balance between the groups with respect to geography and social disadvantage. The balancing also included a random aspect, of 75%, assignation to that arm which would minimize the difference. One EYS randomized to BEST in Wave 2 withdrew from the project.

**TABLE 1:** Early years setting and child participant characteristics.

	All settings		BEST		DLS		
	Wave 1	Wave 2	Wave 1	Wave 2	Wave 1	Wave 2	*p*
*Early years setting*							
*N*	10	10	5	5^a^	5	5	
SES: Ever 6,^b^ *M* (SD)	37.50 (20.31)	38.57 (20.16)	41.58 (21.92)	34.18 (20.95)	33.42 (20.16)	42.08 (21.20)	0.95
Range	11.10–63.50	11.00–64.00	11.10–63.50	11.00–53.70	15.50–60.90	18.50–64.00	
*Children*							
*N*	50	52	21	23	29	29	0.23
No. per setting, *M* (SD)	5 (3.16)	5.2 (1.87)	4.2 (2.17)	4.6 (2.51)	5.8 (4.02)	5.8 (0.84)	
Range	1–10	1–7	2–7	1–7	1–10	5–7	
Gender, %M/F	56/44	60/40	43/57	61/39	66/34	59/41	0.32
Age, *M* (SD)	4;00 (0;03)	3;11 (0;04)	3;10 (0;02)	3;10 (0;03)	4;00 (0;03)	3;11 (0;04)	0.30
Range	3;06–4;05	3;05–4;05	3;07–4;05	3;05–4;04	3;06–4;05	3;05–4;05	
NRDLS comprehension SS, *M* (SD)	79.88 (7.79)	79.90 (9.59)	83.38 (7.53)	82.96 (11.65)	77.34 (7.06)	77.48 (6.87)	0.001
Range	69–95	69–116	69–95	69–116	69–92	69–92	
NRDLS production SS, *M* (SD)	73.62 (5.17)	76.79 (9.02)	74.24 (5.00)	76.61 (9.04)	73.17 (5.33)	76.93 (9.16)	0.78
Range	69–86	69–107	69–85	69–101	69–86	69–107	
Vineland SS,^c^ *M* (SD)	82.68 (6.42)	83.28 (9.85)	83.72 (4.94)	84.43 (10.37)	82.03 (7.19)	81.67 (9.19)	0.22
Range	68–97	69–107	78–97	69–107	68–95	71–103	
Group sizes, *M* (SD)	2.4 (1.1)	2.3 (1.1)	3.0 (0.6)	2.6 (1.3)	2.1 (1.2)	2.1 (1.1)	0.05
Range	1–5	1–5	2–4	1–5	1–5	1–4	
SES IDACI median, IQR	1 (2)	1.5 (3)	2 (4)	2 (3)	1 (2)	1 (3)	0.12
Range							

*Note: p*‐values represent comparisons between intervention arms for each demographic characteristic using *t*‐tests, independent samples median test or Chi‐squared as appropriate. Ever 6, used to calculate pupil premium, is the number of children in a setting who had a recorded period of free school meals in the previous 6 years (Education and Skills Funding Agency 2022); Vineland is a measure of adapted behaviour in three domains: communication, daily living skills and socialization and combine to provide an Adaptive Behaviour Composite (Sparrow et al. 2016); IDACI is a composite index of deprivation for postcode in England which are ranked (Ministry of Housing Communities and Local Government 2019).

NRDL, New Reynell Developmental Language Scales; SES, socio‐economic status; SS, standard scores.

^a^ Ever6 data available for four settings at Wave 1.

^b^ Ever6 data are not available for early years settings, which are not part of the school; one setting was a standalone nursery. This data are provided for 10 settings in Wave 1 and nine for Wave 2.

^c^ *n* = 83.

### 2.3 Recruitment of children

EYS staff were asked to identify children who met the following criteria: age 3;05–4;05; monolingual speaker of English or English as primary language; language development below age‐related expectations based on practitioner judgement and consideration of EYFSP guidance; able to participate in small group learning; no sensorineural hearing impairment, severe visual impairment or diagnosed learning disability. Staff approached parents/carers of children they judged met study eligibility criteria, sharing information and consent forms. Parents/carers were told which arm of the study their child's setting had been allocated to before they signed up for the study. Once consent was obtained, children were blind assessed by RAs (T0) and only included in the interventions if they met the following additional inclusion criteria: demonstrated symbolic play, triadic attention and sufficient attention and turn‐taking ability to participate in small group activities and scored at or below the 16th centile for production and/or comprehension on the New Reynell Language Development Scales (NRDLS) (Edwards et al. 2011).

### 2.4 Measures

Children were assessed by RAs blind to treatment arm allocation for eligibility (T0), before the intervention (T1), immediately after the intervention (T2) and at follow‐up (T3 approximately 9 weeks after T2). The average gap between T1 and the start of intervention for most EYS (*n* = 15) was 3.8 weeks (SD = 1.6; range = 1–7). However, for 5 EYS (3 BEST; 2 A‐DLS), there was a gap of about 11 weeks due to unavoidable staffing changes during the study.

The outcomes were oral language development and communicative participation. At T1, T2 and T3, children were tested on standardized measures of receptive and expressive language and their knowledge of language structures targeted in the interventions, and teachers and parents were asked to report on the children's communicative participation.

- *NRDLS* is a standardized, normed reliable and valid omnibus language assessment that measures young children's comprehension and production abilities yielding SSs (Edwards et al. 2011).
- *BEST Assessment* is a probe designed to monitor progress. Children describe 16 images representing the verbs targeted in the intervention. The images differ across the three assessment time points, assessing the same structures using different noun vocabulary to reduce practice effects. The child's response to the pictures is transcribed and then scored with respect to the proportion of content words and morphology used correctly, with a maximum possible raw score of 115 (McKean et al. 2013).
- *Adapted Derbyshire Language Scheme Rapid Screening Test*: The DLS includes a Rapid Screening Test of children's ability to follow instructions to manipulate toys which contain 1, 2 and 3 information‐carrying words (ICWs) (e.g., show me the cup; put the spoon in the cup; put the pencil under the box) and commands containing ‘and’ in lists and sequences of instructions (Knowles and Masidlover 1982). The child's word level, which is the highest number of ICWs with at least half the test items correct, indicates the starting point for intervention for those receiving A‐DLS. Additional instructions containing 4 ICWs were added (e.g., put the big key on Teddy's plate), combined with scores from 44 items from NRDLS, which assess comprehension of 2, 3 and 4 ICWs, to gain a sensitive measure of change due to intervention. A raw score from the total of 69 items was derived as the outcome.
- *Functional Outcomes in Children Under Six—FOCUS‐34* (Thomas‐Stonell et al. 2012) was completed by parents and teachers to provide a measure of communicative participation in different areas of their lives (home, nursery, playing with friends). FOCUS‐34 has two sections, the first measures how well the child communicates in their daily life, whilst the second measures how much help the child needs to do certain things. The parent or teacher uses a 7‐point scale ranging from ‘not at all like my child’ to ‘exactly like my child’. The FOCUS‐34 does not generate *SSs* but enables the evaluation of change over time and the identification of meaningful clinical change. This is defined as changes in the child's function that are considered to be important to both SLT and parents/carers. A difference in scores pre‐ and post‐intervention >16 is classified as a significant clinical change (SCC).
- *Vineland Adaptive Behaviour Scales*—*Vineland‐3* were completed by the child's teacher at T0, to characterize children's non‐verbal and broader developmental profiles (Sparrow et al. 2016). This questionnaire measures personal and social skills needed for everyday life. Domains include communication, daily living skills, socialization and maladaptive behaviours and combine to provide an Adaptive Behaviour Composite.

### 2.5 Interventions

- *BEST* is usually delivered in small groups (3–6 children) but can be delivered one‐to‐one. A total of 16 sessions of approximately 15‐min duration are delivered twice weekly for 8 weeks. BEST aims to improve children's use and understanding of 2‐, 3‐ and 4‐clause sentences (i.e., 2: The girl is jumping; 3: the boy is eating a banana; 4: the baby is putting the cup on the table) (see Appendix 3 in the Supporting Information section). The intervention exposes children to models of the target sentences in a controlled way, involving both massed and distributed exposure, with controlled variation within the target sentences and controlled contrast between the sentences heard, all presented within a joint action routine. Within each session, children are taken through a two‐phase process three times. In Phase 1 (input with variation), the child hears Verb 1 (e.g., eat) of the target PAS (e.g., Agent + Action + Patient) used 3–6 times with a frame held constant and one slot varied (e.g., The man is eating an *apple*, the man is eating an *orange*, the man is eating a *banana*). Whilst hearing the input, the child sees the actions being completed by the adult with miniature toys. Sign is used alongside speech signalling content (Walker 1987) and morphology (Paget Gorman Signed Speech—(Rowe 1981)). In Phase 2 (output with variation and contrast), the child watches the adult act out an event with the same PAS as Phase 1 but with a contrasting verb, and the child is encouraged to describe what they see and the adult recasts their attempt verbally and with sign (again signalling content (Walker 1987) and morphology (Paget Gorman Signed Speech—(Rowe 1981)). The child is then allowed to act out the event with the toys while the adult again provides a model of the target utterance. This is repeated a number of times, again with a frame held constant and one slot varied. Following each session parents are given a homework booklet containing pictures of the verbs targeted in the session with a range of agents and patients. Parents/carers are encouraged to describe the pictures and so provide repeated input of the target sentences. The child is not expected to repeat or imitate these sentences but is praised and rewarded if they do so spontaneously. A video explaining and demonstrating the homework was made available and parents were texted as reminders after each session with a link to the video. The intervention is described in greater detail in Trebacz et al. (2024) and the manual, intervention and homework resources (McKean et al. 2013) are available from https://research.ncl.ac.uk/lively/interventions/best/.
- *A‐DLS*: The DLS (Knowles and Masidlover 1982) is a flexible syllabus of structured play‐based activities which is individualized to a child's profile of language skills considering both their comprehension and production abilities. The content of and progression through the syllabus is based on typical language development and the work of Bloom (Bloom and Lahey 1978; Bloom et al. 1975) and Brown (Brown 1976; Brown et al. 1981). The scheme includes assessment materials to determine the child's level of abilities, and their intervention starting point, and to monitor their progress. Children's progression is individualized through stages increasing their understanding and use of sentences with 1, 2, 3 and 4 information‐carrying words. Play‐based activities designed to be meaningful to preschool children are used where children are encouraged to verbally direct other children or the teacher. We created an adapted version of DLS (A‐DLS) which could be delivered with high treatment fidelity and reliability in a research context and which matched BEST as closely as possible in terms of dosage and delivery whilst retaining DLS key principles and characteristics. Masidlover, one of the original creators of DLS, created new DLS materials for each activity and provided detailed feedback and advice in the development of the manual and approach. The manual is available at https://www.derbyshire‐language‐scheme.co.uk/AdaptedDLSManual.pdf. The activities and sessions were piloted and refined before the trial commenced. A‐DLS was delivered in small groups with children at either 1–2, 2–3 or 3–4 word level. Homework packs for each activity were developed and provided together with guidance videos for parents (https://research.ncl.ac.uk/lively/interventions/dls/dls/). Parents were sent a text message reminding them about the homework, which specific pack to choose to reinforce the work done in the session and linked to the relevant video where explanations and models were provided. A‐DLS differs from traditional DLS in that children move more rapidly through the range of DLS target sentences and are less individualized in terms of progression and modification of resources. More details of these differences are available on the study website (https://research.ncl.ac.uk/lively/interventions/dls/theadapteddls/).

### 2.6 Treatment fidelity

Detailed manuals, scripts for each session and recording forms were developed for both interventions and a standard set of toy resources and homework materials created. Observational rating scales were used to assess RAs' fidelity to the intervention (see Baker et al. 2024 for further details). Prior to intervention delivery in the trial, RAs were trained in the interventions by C.M. and S.P.; this included video recording their delivery of interventions, reflecting on their fidelity and receiving feedback from S.P. and C.M. using that rating scale. Over the course of the study, fidelity was checked in the same way for all intervention groups in Week 2 of intervention delivery. Due to video recording failure in a small number of sessions, 98.02% of groups were rated. It must be noted that some aspects of delivery were affected by the COVID pandemic. All soft toys were necessarily replaced by toys which could be disinfected and resources were sanitized after each session. The RAs wore gloves, aprons and visors designed for use in paediatric care (i.e., decorated with animals). Children were shown videos to help them prepare for how the RAs would be dressed. RAs used their skills and experience as SLTs to ensure the children were comfortable and to build rapport.

### 2.7 Analysis

All analyses were completed using analyses of covariance (ANCOVAs) to compare outcomes between groups at T2 and T3 adjusting for any group differences which were present in relevant outcomes at T1. Bootstrapping (1000 samples), with replacement, was used to provide standard errors and 95% confidence intervals for the respective regression coefficients. This approach can be used when there are small samples. It repeats the analysis with randomly drawn new samples from the available data set and in so doing creates more robust estimates with associated confidence intervals. Given the small sample size, bootstrapping was preferred over robust standard errors (Mansournia et al. 2020). Multilevel modelling was considered to account for clustering within schools but was rejected due to the small cluster sizes within schools (range = 1–10) and small total sample size (Maas and Hox 2005; McNeish 2014). These analyses were repeated for NRDLS Production and Comprehension SS covarying Wave and Delayed Intervention to assess for potential confounds linked to the pandemic‐related differing experiences of the children receiving interventions in Waves 1 and 2, and for delayed intervention which occurred in five schools. Within‐group analyses over different time periods were conducted with paired *t*‐tests and checked with repeated measures analysis of variance (ANOVA).

## 3 Results

### 3.1 Participants

**FIGURE 1:**
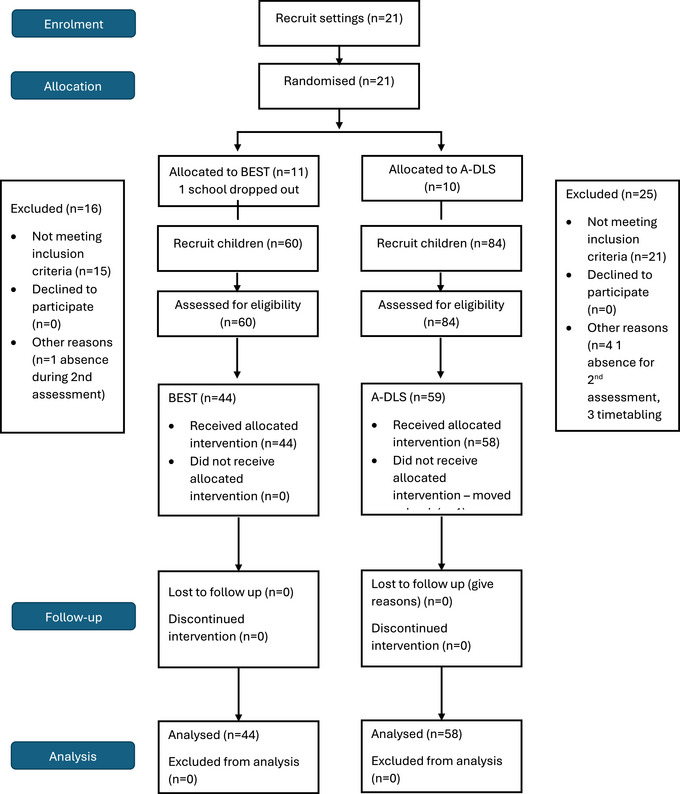
CONSORT diagram showing participant flow through the study.

A total of 178 children were put forward by EY practitioners as children who were not meeting age‐related expectations according to the UK EYFSP and curriculum. Parental consent was given to 144 children who were then assessed for eligibility according to inclusion and exclusion criteria. A total of 103 met those criteria, and one left the study (Figure 1).

A total of 102 children across 20 schools received intervention: 44 receiving BEST and 57 receiving A‐DLS. School and participant demographic characteristics are presented in Table 1. There were no significant differences between intervention groups with respect to EYS SES, numbers per setting, children's gender, age and non‐verbal abilities. There was a significant difference in group sizes across interventions, with smaller groups in the DLS arm due to the greater individualization of the intervention tasks. There was no significant difference between groups at baseline for NRDLS production scores (BEST *M* = 75.5; A‐DLS *M* = 75.1), but there was a significant difference for NRDLS comprehension scores, with higher scores in the BEST group (BEST *M* = 83.2; A‐DLS *M* = 77.4). Analyses reported below adjust for these baseline differences. Using the Indices of Deprivation Affecting Children (IDACI) (Ministry of Housing Communities and Local Government 2019) as a measure of social disadvantage suggests the participants are relatively socially disadvantaged (66% in Quintile 1 (Q1––most disadvantaged 20% in England), 12% in Q2; 7% Q3; 3% Q4; 12% Q5). The median IDACI decile for the children in the A‐DLS arm was lower than the BEST arm; however, this difference was not statistically significant.

### 3.2 Intervention delivery

RAs delivered on average 15.86/16 sessions (SD = 0.34, range = 15–16). These were similar for BEST (*M* = 15.95/16, SD = 0.21, range = 15–16) and A‐DLS (*M* = 15.79/16, SD = 0.41, range = 15–16). Missed sessions were in the main due to COVID pandemic restrictions disrupting EYS provision. Some children did not attend all offered sessions due to EYS absence. Overall children received an average of 13.44/16 sessions (SD = 2.08, range = 6–16). These were similar for BEST (*M* = 13.34/16, SD = 2.29, range = 7–16) and A‐DLS (*M* = 13.51/16, SD = 1.91/16, range = 6–16). Treatment fidelity was high with average percentages on the rating scales (where 100% represents perfect fidelity) 97.82 (SD = 3.84). BEST average fidelity was 99.00 (SD = 1.94) and A‐DLS fidelity was 96.60 (SD = 4.83).

### 3.3 Outcomes

Table 2 presents summary data for each outcome at each data point (T1, T2, T3). There was minimal missing data for the face‐to‐face assessments. However, the FOCUS‐34 returns from parents were very low (T1: 68/102; T2: 49/102; T3: 43/102) and so are not used for further analyses. Returns from teachers were more complete.

**TABLE 2:** Number of completed assessments (*N*), mean (*M*) and standard deviation (SD) scores for the BEST and A‐DLS interventions at preintervention (T1), post‐intervention (T2) and follow‐up (T3), ANCOVA results examining within and between‐group differences with T1 scores as covariate.

											BEST—A‐DLS		Within arm			
	Full sample			BEST Intervention			A‐DLS Intervention				Model		BEST		DLS	
	*N*	*M*	SD	*N*	*M*	SD	*N*	*M*	SD	*d*/*η* ^2^	*F* (d.f.)	*p*	*t* (d.f.)	*p*	*t* (d.f.)	*p*
*NRDLS comprehension SS*																
T1	102	78.89	8.71	44	83.16	9.79	58	77.41	6.91							
T2	100	87.22	12.89	44	88.45	13.23	56	86.25	12.65							
T3	102	90.23	15.3	44	94.89	14.23	58	86.69	15.24							
T1–T2										0.06/0.001	0.103 (1)	0.748	−2.52 (43)	0.016	−5.61 (55)	< 0.001
T2–T3										0.56/0.07	7.593 (1)	**0.007**	−4.17 (43)	< 0.001	−0.56 (55)	0.577
T1–T3										0.26/0.02	1.631 (1)	0.205	−5.81 (43)	< 0.001	−5.30 (57)	< 0.001
*NRDLS production SS*																
T1	102	75.24	7.52	44	75.48	7.41	58	75.05	7.66							
T2	95	84.08	13.09	44	86.16	12.98	51	82.29	13.05							
T3	102	84.98	12.94	44	88.7	13.96	58	82.16	11.44							
T1–T2										0.30/0.02	2.16 (1)	0.145	−6.75 (43)	< 0.001	−5.24 (55)	< 0.001
T2–T3										0.40/0.04	3.64 (1)	0.059	−1.47 (43)	0.149	−0.12 (50)	0.903
T1–T3										0.55/0.07	7.56 (1)	**0.007**	−71 (43)	< 0.001	−5.41 (57)	< 0.001
*BEST assessment*																
T1	98	26.12	16.82	41	29.21	18.70	57	23.89	15.11							
T2	100	57.41	22.49	44	67.93	23.47	56	49.14	17.93							
T3	102	56.82	21.91	44	64.29	21.51	58	51.16	20.63							
T1–T2										0.77/0.13	13.864 (1)	**< 0.001**	−10.24 (40)	< 0.001	−11.33 (54)	< 0.001
T2–T3										0.00/0.00	0.04 (1)	0.841	1.26 (43)	0.215	−0.87 (55)	0.390
T1–T3										0.44/0.05	4.6 (1)	**0.035**	−9.67 (40)	< 0.001	−10.58 (56)	< 0.001
*A‐DLS adapted RST*																
T1	102	47.2	8.08	44	49.61	7.07	58	45.36	8.38							
T2	100	51.45	7.22	44	53.2	4.91	56	50.07	8.41							
T3	102	52.99	5.98	44	54.27	5.17	58	52.02	6.4							
T1–T2										0.21/0.01	1.09 (1)	0.299	−4.05 (43)	< 0.001	−3.89 (55)	< 0.001
T2–T3										0.13/0.004	0.38 (1)	0.541	−1.79 (43)	0.08	−2.17 (55)	0.034
T1–T3										0.14/0.01	0.48 (1)	0.489	−4.68 (43)	< 0.001	−6.55 (55)	< 0.001
*FOCUS‐34 score*																
T1	98	132.47	44.39	44	132.89	38.17	54	132.13	49.24							
T2	93	166.22	39.37	40	165.73	35.64	53	166.58	42.31							
T3	77	162.57	41.21	23	154.61	41.6	54	165.96	40.96							
T1–T2										0.17/.01	0.58 (1)	0.447	−6.51 (39)	< 0.001	−6.98 (50)	< 0.001
FOCUS‐34 SCC		% SCC			% SCC		*N*	% SCC			Chi^2^	*p*				
T1–T2	93	66			73			61			1.50	0.22				

*Note*: A‐DLS, Adapted Derbyshire Language Scheme; BEST, Building Early Sentences Therapy; FOCUS‐34: functional outcomes in children under 6; NRDLS, New Reynell Developmental Language Scales; RST, Rapid Screening Test; SCC, significant clinical change; SS, standard score.

Results of the ANCOVA analyses are presented in Table 2 and highlighted in bold where significant differences between time points and between groups were found. Group comparisons at T2 and T3 were adjusted for T1 scores. The following interprets those results with reference to each research question. ESs are also presented in Table 2. Cohen's *d* was calculated from ANCOVA partial eta squared using an ES converter (MRC Cognition and Brain Sciences Unit 2009) based on formulae from Cohen (1988, 281, 284, 285). ESs are interpreted below with reference to Coe et al.’s criteria for use in educational interventions which provide an estimate of months progress gained from the intervention (Coe et al. 2013) (see Appendix 5 in the Supporting Information section).

*RQ 1  Which intervention is most effective?*

**FIGURE 2:**
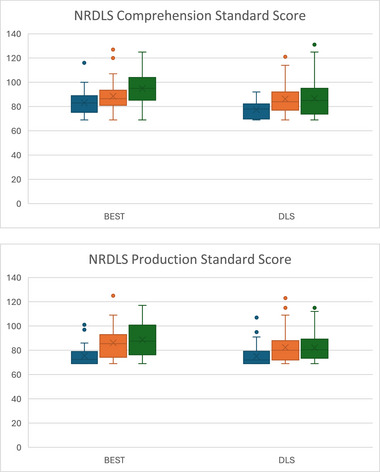
Box and whisker plots of NRDLS comprehension and production standard score at T1, T2 and T3 for each intervention arm.

**FIGURE 3:**
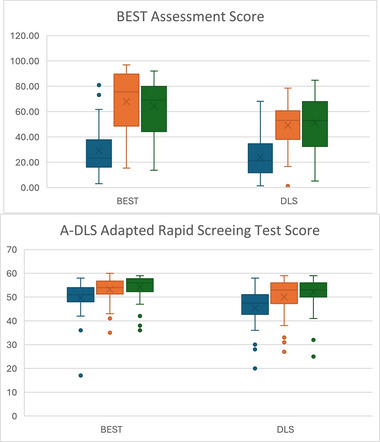
Box and whisker plots of BEST assessment and A‐DLS adapted RST scores at T1, T2 and T3 for each intervention arm.

**FIGURE 4:**
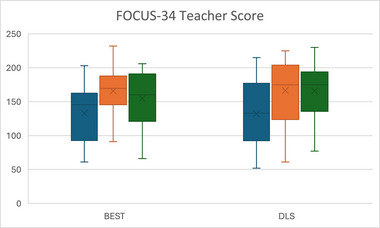
Box and whisker plot of FOCUS‐34 at T1, T2 and T3 for each intervention arm.

BEST was associated with significantly greater gains than A‐DLS in SS in NRDLS comprehension between T2 and T3 with a high ES (0.56), and production between T1 and T3, again with high ES (0.55). Gains between T2 and T3 for production SS did not reach significance (*p* = 0.059; moderate ES = 0.40); however, when the confound of wave is included, this comparison is significant. BEST was also associated with greater gains in BEST‐targeted sentences at T2 and T3 with very high (0.78) and high (0.44) ESs, respectively. There were no other significant differences between the treatment arms (Figures 2, 3, 4 and Table 2).

*RQ 2 Do interventions differ in the degree to which benefits transfer to non‐targeted language structures and/or communicative participation?*

Both intervention arms made significant improvements from T1 to T2 in all outcomes: NRDLS Production SS, NRDLS Comprehension SS, BEST Assessment scores, A‐DLS Adapted RST and FOCUS‐34. These benefits were maintained at T3 such that significant improvements from T1 to T3 were also present for all measures.

Improvements in NRDLS SS, which are corrected for age, potentially represent catch‐up growth in language with changes in average SSs from T1 to T2 of 8 for comprehension (BEST = 5; A‐DLS = 9) and 9 for production (BEST = 10; A‐DLS = 7); and between T1 and T3 of 11 for comprehension (BEST = 12; A‐DLS = 9) and 9 for production (BEST = 13; A‐DLS = 7). The FOCUS‐34 classifies a change in score of >16 as a SCC in communicative participation. The average change between T1 and T2 was 33 (BEST = 32; DLS = 34) with 66% of children reaching the SCC threshold (BEST = 73%; A‐DLS: –61%). At T3, there was substantial missing data and so T1 to T3 changes are not considered further (Table 2).

Results on NRDLS and FOCUS‐34 represent non‐targeted language structures and communicative participation, respectively. BEST was associated with significantly greater gains in both NRDLS comprehension and production than for A‐DLS. There were no significant differences between groups in communicative participation (Figures 2, 3, 4 and Table 2).

*RQ 3 Do interventions differ in the degree to which language abilities continue to improve after the intervention is complete?*

BEST intervention was associated with significantly greater gains between T2 and T3 than A‐DLS in NRDLS comprehension SS and production SS in an adjusted model (see below). There were no significant differences between groups on T2 to T3 gains for the measures of targeted language structures (BEST Assessment and A‐DLS Adapted RST). However, the A‐DLS was associated with significant progress from T2–T3 in the A‐DLS Adapted Rapid Screening Test which was not present for those receiving the BEST intervention.

### 3.4 Potential confounds

Finally, models examining NRDLS comprehension and production SS were rerun adjusted for the potential confounds of wave and delayed intervention. Due to the small sample size, these were completed in separate models. Results are presented in Appendix 4 in the Supporting Information section. No substantive differences in the pattern of results were found although the difference between BEST and A‐DLS for NRDLS T2–T3 gains in production SS becomes significant when the wave was covaried.

## 4 Discussion

Both interventions were associated with significant improvements in all outcomes, including in SSs, although in the absence of a no‐treatment control, such changes alone cannot be interpreted as proof of efficacy. However, comparison between two active intervention arms provides a highly stringent test of efficacy should, as in this case, one arm yields greater gains than the other.

Most of the study hypotheses were supported. As predicted, BEST was associated with greater gains in children's production with high ESs (*d* = 0.55); however, we did not find the predicted greater gains for comprehension for A‐DLS, where the interventions appeared to bring equivalent benefits. Due to the hypothesized promotion of abstract representations allowing knowledge to be generalized, we expected BEST would bring greater benefits in non‐targeted structures and promote greater gains after the intervention. This was supported by greater gains in production SSs and by the pattern of accelerated progress after the intervention for both production and comprehension scores. That is, BEST was associated with greater gains after the intervention was complete (T2–T3) with moderate to high ES (comprehension *d* = 0.56; production *d* = 0.40). Raw score gains in targeted language structures (BEST Assessment and A‐DLS Adapted RST) between baseline and outcome (T1–T2) favoured BEST with a very high ES for BEST Assessment scores (*d* = 0.77) and no significant differences were found between interventions in the A‐DLS Adapted RST scores. BEST is, therefore, effective in improving the production of sentence structures targeted in the intervention and in promoting generalization to non‐targeted language structures. The interventions are equally effective at improving comprehension.

The majority of children across interventions (66%) made gains in their communicative participation which reached the threshold for a clinically significant change and there were significant changes for both interventions between T1 and T2 in this outcome measure. There were no significant group differences in this outcome. These gains in both language SS and communicative participation outcomes are very encouraging particularly given the low dosage of sixteen 15‐min small‐group interventions over 8 weeks.

The ESs described above are interpreted with reference to Coe et al.’s criteria for use in educational interventions (Coe et al. 2013) which were developed for comparison between an intervention and TAU control. This study, comparing two active interventions, therefore provides a highly conservative estimate of ES yet we find larger effects than other targeted small‐group interventions with higher dosages (Bleses et al. 2018; West et al. 2024). These positive findings in the relatively socially disadvantaged sample in the study are particularly encouraging given that some pre‐school interventions can widen rather than narrow inequalities (McKean and Reilly 2023).

The lack of a non‐intervention control due to changes to the study following COVID restrictions makes it difficult to be sure that A‐DLS brings benefits over and above the usual EYS practice. However, previous research would suggest this is highly likely (Broomfield and Dodd 2011). Creating change in non‐targeted language structures is vital for effective and efficient intervention. Greater gains for the BEST intervention in production SS, that is language structures not targeted in the intervention, prove BEST's efficacy, supporting the findings of Trebacz et al. (2024) but in a more rigorous study methodology. Furthermore, these findings suggest it may be more efficient than other interventions although this requires further research.

Faster progress after intervention supports the hypothesis that the active ingredients in BEST, based on usage‐based theory, do promote the development of abstract representations of PAS, supporting generalization and accelerating language learning. Longer term follow‐up is needed to test how long such benefits might be present for a child; however, it demonstrates the significant promise of interventions designed with reference to usage‐based theory and which precisely manipulate hypothesized cognitive and linguistic active ingredients to promote change.

### 4.1 Strengths and limitations

This pre‐registered (ISRCTN10974028) cluster randomized controlled trial following CONSORT guidance for conduct and reporting represents a rigorous evaluation of the relative efficacy of BEST and A‐DLS including randomization, blinding, extensive treatment fidelity strategies and minimal dropout and missing data (Campbell et al. 2012). The effects of COVID meant the study had a smaller overall sample size than originally planned. The sample in each treatment arm did not reach the target of 65 derived from power calculations (A‐DLS = 58; BEST = 45); however, data were maximized through high retention and data completeness. The smaller sample size could inflate the ESs found.

We acknowledge that the analysis performed does not take into account the non‐independence in the data (child within school) and that a multilevel approach would have been preferable. This approach was not adopted given the number of schools and children in each school (in some cases only a single child) (Maas and Hox 2005; McNeish 2014). Bootstrapping was used with no substantive differences obtained.

The A‐DLS Adapted RST used to assess progress in target structures did contain some structures not targeted in the A‐DLS for each child and so perhaps could underestimate the progress made in this arm.

Removing the TAU arm due to ethical concerns made it harder to draw conclusions about the efficacy of whichever intervention had a smaller effect, in this case, A‐DLS. Furthermore, the adaption of A‐DLS makes it difficult to generalize our findings to standard DLS. However, this head‐to‐head approach, matching interventions with respect to dosage, delivery context, fidelity and so forth, enables us to rule out general therapy effects. We can be sure that it is the specific qualities of BEST that affect change in production scores over and above small group play activities.

## 5 Conclusions

Services need access to multiple interventions with proven efficacy to choose approaches that best fit the needs of the populations they serve, align with the constraints of service provision, and bring lasting benefits to children. This study provides evidence from a rigorous RCT that it is possible to bring about moderate to high language gains for preschool children, including those from low socio‐economic backgrounds, with a low dosage intervention (*d* = 0.44 BEST assessment raw scores; *d* = 0.55 NRDLS production SS). Our findings using a head‐to‐head comparator likely underestimate these effects, which Coe et al.’s approach suggests are equivalent to 5 and 7 months progress, respectively. Given the intense pressures on early years and SLT services, it is essential that effective interventions are identified which are feasible for implementation within such constraints. BEST may offer such an approach for some children. In terms of profiles of need this study shows that children with production difficulties benefit from BEST. Previous study findings suggest DLS improves comprehension. It is, therefore, possible that BEST and DLS both bring benefits in this domain, but further research is required to test this assumption regarding BEST.

The accelerated progress after intervention underscores the significant potential of interventions designed with reference to usage‐based theory and which precisely manipulate language exposure to leverage cognitive mechanisms to promote language learning and abstract knowledge. We recommend that further research is conducted to examine whether longer term gains persist after intervention, the wider potential of the application of usage‐based theory to language interventions and health economic evaluation to consider both the efficacy and efficiency of early interventions.

## Conflicts of Interest

BEST materials are freely available for download from https://research.ncl.ac.uk/lively/interventions/best/. No profits are made by the authors. The Adapted Derbyshire Language Scheme (A‐DLS) manual is available on the DLS website. M.M. receives royalties from the sale of DLS materials purchased on the site.

## Supporting information



Appendix 1

Appendix 2

Appendix 3

Appendix 4

Appendix 5

Appendix 6

## Data Availability

Data are deposited in the Newcastle University data repository and are openly available for use by other researchers at https://data.ncl.ac.uk/.
