# Supplementary material for: A Cluster Randomized Controlled Trial Comparing the Efficacy of Pre‐School Language Interventions—Building Early Sentences Therapy and an Adapted Derbyshire Language Scheme
Source: Int J Lang Commun Disord. 2025 Apr 26;60(3):e70036. doi: 10.1111/1460-6984.70036 (PMC12032828; doi:10.1111/1460-6984.70036)
Supplement: Supplementary file 1 — Appendix 1 [file JLCD-60-0-s002.docx]

Appendix 1 - Stages of multi-word utterance development, relevant cognitive mechanisms, and their use as active ingredients in BEST (McKean et al., 2013; Tomasello, 2003). Reproduced from Trebacz, A., McKean, C., Stringer, H. & Pert, S. (2024) Piloting building early sentences therapy for pre-school children with low language abilities: An examination of efficacy and the role of sign as an active ingredient. *International Journal of Language & Communication Disorders*, 59, 1128–1151. <https://doi.org/10.1111/1460-6984.12980>

| Stage | Relevant cognitive mechanisms | | |
| --- | --- | --- | --- |
|  | Name | Description | Relevant BEST ‘active ingredient’ |
| 1. *Frozen Phases*   Rote learned, and therefore inflexible, utterances paired with a pragmatic function and a communicative context/cultural routine. The child cannot combine the elements of the structure productively with other words.  e.g. “*eat it*” bound to a meal time social routine or “*shoes off”* bound to an undressing routine | *Intention reading* | To create the form-function mapping required for the development of frozen phrases, the child must ‘read’ the communicative intentions of the person from whom they are learning the phrase. The child’s ability to read the intentions of others within the scaffolding of joint attentional frames (Tomasello, 2003 p.21). | A structured and repetitive ‘joint action routine’ is established, creating a joint attentional frame between the child and the adult, which ‘scaffolds’ the child’s ability to infer the communicative intention of the utterances they hear (i.e. describing an event within a play activity). Hence the child quickly becomes able to infer the communicative intentions of the adult at the level of the attentional frame (which objects and actions are we both attending to and what is the global purpose of this joint attention); and so is supported to make such inferences at the level of the individual communicative acts within that frame (which objects and actions is the adult referring to with a specific utterance) (Tomasello, 2003). |
|  | *Cultural learning* | A process by which young children learn through imitation (and later through instructed and collaborative learning) of others in their social group (Tomasello, 2003 p.290). The child must not only mirror the communicative behaviour of the adult, but also understand that the roles within the triadic attentional frame (e.g. adult - child - object/action) reverse when they imitate the adult. Through this process, the child comprehends that when they are the speaker, imitating the communication of the adult, the communicative intention of the adult that was directed to the child instead becomes directed to the adult (Tomasello, 2003 p.26). | Role reversal is used within the ‘joint action routine’ to promote cultural learning and hence the creation of symbolic linguistic representations (Tomasello, 2003). |
| 1. *Lexically specific constructions*   Partially productive/flexible utterances with a ‘slot and frame’ construction where only one element can vary (e.g. “*X fall down*” or “*I’m ACTIONing it*”). ‘Frame’ categories might be:  “X fall down” – the category of events in which animate or inanimate objects unintentionally drop to a lower place  “I’m ACTIONing it” – the category of events in which the child is performing an action on an object.  ‘Slot’ categories might be:  X fall down – where X is the category of animate or inanimate objects which can fall  I’m ACTIONing it – where ACTION is the category of the things I can do to objects  Eat X – where X is the category of objects which can be eaten. | *Schemat-isation* | A general cognitive strategy that facilitates the identification of rules and patterns or *schemas*, or within the child’s environment, supporting them to rely on mental abstractions (Piaget, 1952). In the case of communication, multiple exposures to the same utterances where one component is varied across exposures (e.g. X fall down, where X is *the girl* and then *the boy* and then *the teddy)* enable children to create rules or *schemas* which represent the aspects of the construction that remain the same across iterations (the ‘frame’), and which components vary across iterations (the ‘slot’, in this case X) (Gomez, 2002; Tomasello, 2003 p.122). | The cognitive processes of *schematisation* and *categorisation* both depend on the quantity and distribution of types and tokens within the input heard by the child.  BEST provides multiple presentations of highly similar exemplar sentences in which one element is varied systematically (Gomez, 2002; Tomasello, 2003).  (e.g. The baby is laughing; The woman is laughing; The girl is laughing; The teddy is laughing). |
|  | *Categori-sation* | In order to effectively use the ‘slot and frame’ constructions emerging from the process of schematisation, children must also form mental *categories* of which items can be put into each ‘slot’. At the stage of *lexically specific constructions*, the child’s categories are still functional and relatively concrete (e.g. in the construction “*X fall down*, X might consist of ‘animate objects which involuntarily move from a high place to a lower place’, and the category ACTION in “*He’s ACTIONing it*” would consist of ‘actions ‘he’ can perform’ (Tomasello, 2003 p.124). |  |
| 1. *Abstract constructions*   Flexible, abstract representations allowing children to use any relevant lexical items in the appropriate role in the sentence and so use the sentence structures productively.  Analogy across functional relationships supports the creation of semantic categories (e.g. AGENT, PATIENT) and constructions (e.g. AGENT + ACTION + PATIENT)  Analogy across construction form (but not function) (e.g. ***The*** *girl like****s*** *cake;* ***The*** *rabbit eat****s*** *lettuce)* results in the construction of syntactic categories (e.g. VERB, OBJECT) and constructions (e.g. SUBJECT + VERB + OBJECT). | *Analogy* | Children identify patterns and commonalities between phenomena, in the case of linguistic abstract constructions, the shared *functional* relationships between items. Such categories are analogous because the functional relationships are the same across constructions, e.g. *‘The* ***A*** *is* ***B****ing the* ***C****’* is analogous to ‘The ***D*** *is* ***E****ing the* ***F****’*  (Tomasello, 2003 p163).  In the above example, **A** and **D** are doing the action, **B** and **E** are actions, and **C** and **F** are the recipients of the actions. “When an analogy is made the objects involved are effaced; the only identity they retain is their relational structure” (Tomasello, 2003 p.164). | Repeated exposure to sentence construction pairs that have the same predicate argument structure but contrasting verbs (e.g. The teddy is eating the apple; The man is washing the apple) provides children with multiple opportunities to identify the similarities in functional relationships and abstract semantic *categories* (e.g. AGENT, ACTION, PATIENT) and semantic *constructions* (e.g. AGENT + ACTION + PATIENT) (Tomasello, 2003).  For each sentence construction pairing the items in each argument structure role are non-overlapping sets, providing a level of consistency thought to facilitate analogy (McKean et al., 2013) (e.g. AGENTS are never PATIENTS and vice versa).  The use of toys to act out the target sentences support the identification of predicate argument structure roles (e.g. making distinctions between agent and patient more tangible).  Input rotates through the different constructions targeted by BEST. This results in distributed exposure to a range of constructions across which the child can find analogies (Ambridge et al., 2006).  For each sentence construction pairing the morphological frame remains constant (e.g. **The** boy **is** jumping; **The** woman **is** sitt**ing; The** X **is** Y**ing** **the** Z) providing an additional structural cue regarding the similarity between constructions (Tomasello, 2003).  The use of a signing system which marks both lexical items and grammatical morphology. The marking of these items drives pattern finding and thence analogy, supporting children with language difficulties to create abstract representations of predicate argument structure that might otherwise be difficult due to phonological and morphological processing difficulties. Sign also supports semantic mapping and reduces processing load, rendering cues in the input more accessible (Chiat, 2001; Leonard, 2007; Tomasello, 2003; Rowe, 1981; Walker & Armfield, 1981). |
| All stages | *Mapping* | Establishing a representation in memory of a new meaning-construction pairing which is essential for learning words and early multi-word constructions and their corresponding meanings (Hirsh-Pasek et al., 2000). | Many repetitions of the same and similar constructions are provided alongside visual referents (toys and signs) to facilitate mapping which often requires more exposures for children with language difficulties than their typically developing peers. Other verbal input is avoided (Riches et al., 2005). |
| All stages | *Retention* | The formation of robust representations of newly learned constructions in long-term memory for future retrieval (Leonard et al., 2020). | Exposure to constructions is distributed over multiple sessions to leverage spacing effects thought to facilitate long term retention of learning (Riches et al., 2005).  Multiple opportunities to use the target construction expressively, facilitating long term retention (Frizelle & McKean, 2022). |
