# Supplementary material for: A Cluster Randomized Controlled Trial Comparing the Efficacy of Pre‐School Language Interventions—Building Early Sentences Therapy and an Adapted Derbyshire Language Scheme
Source: Int J Lang Commun Disord. 2025 Apr 26;60(3):e70036. doi: 10.1111/1460-6984.70036 (PMC12032828; doi:10.1111/1460-6984.70036)
Supplement: Supplementary file 2 — Appendix 2 [file JLCD-60-0-s004.docx]

Appendix 2 CONSORT CHECKLIST

**Tables**

on 29 April 2024 by guest. Protected by copyright.

[http://www.bmj.com](http://www.bmj.com/)

[/](http://www.bmj.com/)

BMJ: first published as 10.1136/bmj.e5661 on 4 September 2012. Downloaded from

| **Table 1\| CONSORT 2010 checklist of information to include when reporting a cluster randomised trial** | | |  |
| --- | --- | --- | --- |
|  |  | | **Page** |
| **Section/topic and item No** | **Standard checklist item Extension for cluster designs** | | **No*** |
| **Title and abstract** |  | |  |
| 1a | Identification as a randomised trial in the title Identification as a cluster randomised trial in the title | | 2 |
| 1b | Structured summary of trial design, methods, results, and conclusions See table 2 (for specific guidance see CONSORT for abstracts)^1112^ | | 2 |
| **Introduction** |  | |  |
| Background and objectives: |  | |  |
| 2a | Scientific background and explanation of rationale Rationale for using a cluster design | | 5 |
| 2b | Specific objectives or hypotheses Whether objectives pertain to the cluster level, the individual participant level, or both | | 4 |
| **Methods** |  | |  |
| Trial design: |  | |  |
| 3a | Description of trial design (such as parallel, factorial) including Definition of cluster and description of how the design  allocation ratio features apply to the clusters | | 5 |
| 3b | Important changes to methods after trial commencement (such as eligibility criteria), with reasons | | 6 |
| Participants: |  | |  |
| 4a | Eligibility criteria for participants Eligibility criteria for clusters | | 5, 6 |
| 4b | Settings and locations where the data were collected | | 5-6 |
| Interventions: |  | |  |
| 5 | The interventions for each group with sufficient details to allow Whether interventions pertain to the cluster level, the replication, including how and when they were actually administered individual participant level, or both | | 5. 8-9 |
| Outcomes: |  | |  |
| 6a | Completely defined prespecified primary and secondary outcome Whether outcome measures pertain to the cluster measures, including how and when they were assessed level, the individual participant level, or both | | 7-8 |
| 6b | Any changes to trial outcomes after the trial commenced, with reasons | | 14 |
| Sample size: |  | |  |
| 7a | How sample size was determined Method of calculation, number of clusters(s) (and  whether equal or unequal cluster sizes are assumed), cluster size, a coefficient of intracluster correlation (ICC or *k*), and an indication of its uncertainty | | 5 |
| 7b | When applicable, explanation of any interim analyses and stopping guidelines | | N/A |
| **Randomisation** |  |  |  |
| Sequence generation: |  |  |  |
| 8a | Method used to generate the random allocation sequence |  | 6 |
| 8b | Type of randomisation; details of any restriction (such as blocking and block size) | Details of stratification or matching if used | 6 |
| Allocation concealment mechanism: |  |  |  |
| 9 | Mechanism used to implement the random allocation sequence (such as sequentially numbered containers), describing any steps taken to conceal the sequence until interventions were assigned | Specification that allocation was based on clusters rather than individuals and whether allocation concealment (if any) was at the cluster level, the individual participant level, or both | 6 |
| Implementation: |  |  |  |
| 10 | Who generated the random allocation sequence, who enrolled participants, and who assigned participants to interventions | Replaced by 10a, 10b, and 10c | 6 |
| 10a |  | Who generated the random allocation sequence, who enrolled clusters, and who assigned clusters to interventions | 6 |

**Table 1 (continued)**

on 29 April 2024 by guest. Protected by copyright.

[http://www.bmj.com](http://www.bmj.com/)

[/](http://www.bmj.com/)

BMJ: first published as 10.1136/bmj.e5661 on 4 September 2012. Downloaded from

|  |  |  | **Page** |
| --- | --- | --- | --- |
| **Section/topic and item No** | **Standard checklist item** | **Extension for cluster designs** | **No*** |
| 10b |  | Mechanism by which individual participants were included in clusters for the purposes of the trial (such as complete enumeration, random sampling) | 6 |
| 10c |  | From whom consent was sought (representatives of the cluster, or individual cluster members, or both) and whether consent was sought before or after randomisation | 6 |
| Blinding: |  |  |  |
| 11a | If done, who was blinded after assignment to interventions (for example, participants, care providers, those assessing outcomes) and how |  | 7 |
| 11b | If relevant, description of the similarity of interventions |  | 9. |
| Statistical methods: |  |  |  |
| 12a | Statistical methods used to compare groups for primary and secondary outcomes | How clustering was taken into account | 11, 17 |
| 12b | Methods for additional analyses, such as subgroup analyses and adjusted analyses |  | 7 |
| **Results** |  |  |  |
| Participant flow (a diagram is strongly recommended): | |  |  |
| 13a | For each group, the numbers of participants who were randomly assigned, received intended treatment, and were analysed for the primary outcome | For each group, the numbers of clusters that were randomly assigned, received intended treatment, and  were analysed for the primary outcome | Fig 1 |
| 13b | For each group, losses and exclusions after randomisation, together For each group, losses and exclusions for both  with reasons clusters and individual cluster members | | Fig 1 |
| Recruitment: |  |  |  |
| 14a | Dates defining the periods of recruitment and follow-up |  | 7 |
| 14b | Why the trial ended or was stopped |  | N/A |
| Baseline data: |  |  |  |
| 15 | A table showing baseline demographic and clinical characteristics for each group | Baseline characteristics for the individual and cluster levels as applicable for each group | Table 1 |
| Numbers analysed: |  |  |  |
| 16 | For each group, number of participants (denominator) included in each analysis and whether the analysis was by original assigned groups | For each group, number of clusters included in each analysis | Table 2 |
| Outcomes and estimation: |  |  |  |
| 17a | For each primary and secondary outcome, results for each group, and the estimated effect size and its precision (such as 95% confidence interval) | Results at the individual or cluster level as applicable and a coefficient of intracluster correlation (ICC or *k*) for each primary outcome | Table 2, S3 |
| 17b | For binary outcomes, presentation of both absolute and relative effect sizes is recommended |  | N/A |
| Ancillary analyses: |  |  |  |
| 18 | Results of any other analyses performed, including subgroup analyses and adjusted analyses, distinguishing prespecified from exploratory |  | S3 |
| Harms: |  |  |  |
| 19 | All important harms or unintended effects in each group (for specific guidance see CONSORT for harms106) | |  |
| **Discussion** |  | |  |
| Limitations: |  | |  |
| 20 | Trial limitations, addressing sources of potential bias, imprecision, and, if relevant, multiplicity of analyses | | 16-17 |
| Generalisability: |  | |  |
| 21 | Generalisability (external validity, applicability) of the trial findings Generalisability to clusters and/or individual participants (as relevant) | | - |

**Table 1 (continued)**

on 29 April 2024 by guest. Protected by copyright.

[http://www.bmj.com](http://www.bmj.com/)

[/](http://www.bmj.com/)

BMJ: first published as 10.1136/bmj.e5661 on 4 September 2012. Downloaded from

|  |  |  | **Page** |
| --- | --- | --- | --- |
| **Section/topic and item No** | **Standard checklist item** | **Extension for cluster designs** | **No*** |
| Interpretation: |  |  |  |
| 22 | Interpretation consistent with results, balancing benefits and harms, and considering other relevant evidence |  | 15-17 |
| **Other information** |  |  |  |
| Registration: |  |  |  |
| 23 | Registration number and name of trial registry |  | 1, 5, 16 |
| Protocol: |  |  |  |
| 24 | Where the full trial protocol can be accessed, if available |  | N/A |
| Funding: |  |  |  |
| 25 | Sources of funding and other support (such as supply of drugs), role of funders |  | Acknowledgements |
| *Page numbers optional depending on journal requirements. | |  |  |
