# Supplementary material for: A Cluster Randomized Controlled Trial Comparing the Efficacy of Pre‐School Language Interventions—Building Early Sentences Therapy and an Adapted Derbyshire Language Scheme
Source: Int J Lang Commun Disord. 2025 Apr 26;60(3):e70036. doi: 10.1111/1460-6984.70036 (PMC12032828; doi:10.1111/1460-6984.70036)
Supplement: Supplementary file 3 — Appendix 3 [file JLCD-60-0-s005.docx]

Appendix 3

*Verbs and Predicate argument structures targeted by the BEST program and the use of Contrast and Variation in those structures*

| No. of arguments | Set | Argument Structure | Input | Output |
| --- | --- | --- | --- | --- |
| 1 | A | Agent^2^ + Action^1^ | laughing | sitting |
| 1 | B | Agent^2^ + Action^1^ | jumping | walking |
| 2 | C | Agent^1^ + Action^1^ + Patient^2^ | eating | washing |
| 2 | D | Agent^1^ + Action^1^ + Patient^2^ | riding | smelling |
| 2 | E | Agent^1^ + Action^1^ + Patient^2(A)^ | kissing | hugging |
| 2 | F | Agent^1^ + Action^1^ + Patient^2^ | kicking | brushing |
| 3 | G | Agent^1^ + Action + Patient^2^ + Locative^1^ | putting | putting |
| 3 | H | Agent^1^ + Action + Patient^1^ + Locative^2^ | pouring | pouring |
| 3 | I | Agent^1^ + Action^1^ + Patient^2(A)^ + Locative^2(B)^ | putting | pouring |
| 3 | J | Agent^1^ + Action + Patient^2^ + Benefactive^1^ | giving | giving |
| 3 | K | Agent^1^ + Action + Patient^1^ + Benefactive^2^ | throwing | throwing |
| 3 | L | Agent^1^ + Action^1^ + Patient^2(A)^ + Benefactive^2(B)^ | giving | throwing |

1- Contrast between Input & Output; 2- Variation within Input and Output; 2(A)- Variation within Input only; 2(B)- Variation within Input only
