# Supplementary material for: A Cluster Randomized Controlled Trial Comparing the Efficacy of Pre‐School Language Interventions—Building Early Sentences Therapy and an Adapted Derbyshire Language Scheme
Source: Int J Lang Commun Disord. 2025 Apr 26;60(3):e70036. doi: 10.1111/1460-6984.70036 (PMC12032828; doi:10.1111/1460-6984.70036)
Supplement: Supplementary file 4 — Appendix 4 [file JLCD-60-0-s006.docx]

Appendix 4

Results of regression analyses for NRDLS production and comprehension scores unadjusted and adjusted for potential confounds, data wave and delayed intervention

|  | T1 - T2 | | | | T2 - T3 | | | | | | T1 - T3 | | | |
| --- | --- | --- | --- | --- | --- | --- | --- | --- | --- | --- | --- | --- | --- | --- |
|  | *B* | LL | UL | Sig | | *B* | LL | UL | Sig | *B* | | LL | UL | *p* |
| NRDLS Comprehension SS |  |  |  |  | |  |  |  |  |  | |  |  |  |
| Treatment arm | -0.83 | -5.96 | 4.30 | 0.748 | | 6.01 | 1.68 | 10.34 | **0.007** | 3.60 | | -1.99 | 9.19 | 0.205 |
| Adjusted for wave |  |  |  |  | |  |  |  |  |  | |  |  |  |
| Arm group | -0.92 | -5.75 | 3.92 | 0.708 | | 6.07 | 1.76 | 10.39 | **0.006** | 3.36 | | -1.88 | 8.61 | 0.206 |
| Wave | 8.36 | 3.81 | 12.91 | **0.000** | | 2.85 | -1.66 | 7.37 | 0.213 | 9.49 | | 4.58 | 14.40 | **0.000** |
| Adjusted for Delayed Intervention |  |  |  |  | |  |  |  |  |  | |  |  |  |
| Arm group | -0.83 | -5.99 | 4.34 | 0.751 | | 5.80 | 1.54 | 10.07 | **0.008** | 3.71 | | -1.88 | 9.31 | 0.191 |
| DI | 0.15 | -6.47 | 6.77 | 0.964 | | 5.57 | 0.04 | 11.10 | **0.048** | 3.79 | | -3.42 | 10.99 | 0.299 |
| NRDLS Production SS |  |  |  |  | |  |  |  |  |  | |  |  |  |
| Arm group | 3.15 | -1.11 | 7.41 | 0.145 | | 3.57 | -0.14 | 7.29 | 0.059 | 6.24 | | 1.74 | 10.73 | **0.007** |
| Adjusted for Wave |  |  |  |  | |  |  |  |  |  | |  |  |  |
| Arm group | 3.26 | -1.02 | 7.53 | 0.134 | | 4.03 | 0.47 | 7.58 | **0.027** | 6.12 | | 1.81 | 10.43 | **0.006** |
| Wave | 1.82 | -2.59 | 6.23 | 0.415 | | 5.80 | 2.20 | 9.40 | **0.002** | 6.98 | | 2.61 | 11.34 | **0.002** |
| Adjusted for delayed intervention |  |  |  |  | |  |  |  |  |  | |  |  |  |
| Arm group | 3.17 | -1.11 | 7.45 | 0.145 | | 3.54 | -0.12 | 7.20 | 0.058 | 6.04 | | 1.56 | 10.52 | **0.009** |
| Delayed Intervention | -0.86 | -6.62 | 4.89 | 0.767 | | 4.73 | 0.05 | 9.41 | **0.048** | 4.59 | | -1.53 | 10.71 | 0.140 |

*Key: NRDLS: New Reynell Developmental Language Scales; SS: Standard Score;*

Results of Bootstrapped regression analyses for NRDLS production and comprehension scores unadjusted and adjusted for potential confounds, data wave and delayed intervention

|  | T1 - T2 | | | | T2 - T3 | | | | T1 - T3 | | | |
| --- | --- | --- | --- | --- | --- | --- | --- | --- | --- | --- | --- | --- |
|  | *B* | LL | UL | Sig | *B* | LL | UL | Sig | *B* | LL | UL | *p* |
| NRDLS Comprehension SS |  |  |  |  |  |  |  |  |  |  |  |  |
| Arm group | -0.83 | -5.92 | 4.97 | 0.744 | 6.01 | 1.76 | 10.18 | **0.009** | 3.60 | -1.98 | 9.07 | 0.212 |
| Adjusted for wave |  |  |  |  |  |  |  |  |  |  |  |  |
| Arm group | -0.92 | -5.78 | 3.90 | 0.695 | 6.07 | 1.97 | 10.50 | **0.005** | 3.36 | -2.47 | 8.76 | 0.242 |
| Wave | 8.36 | 4.37 | 12.96 | **0.001** | 2.85 | -1.50 | 7.11 | 0.185 | 9.49 | 4.86 | 14.23 | **0.001** |
| Adjusted for Delayed Intervention |  |  |  |  |  |  |  |  |  |  |  |  |
| Arm group | -0.83 | -5.85 | 4.58 | 0.745 | 5.80 | 1.70 | 10.09 | **0.007** | 3.71 | -2.03 | 9.61 | 0.227 |
| DI | 0.15 | -5.98 | 6.12 | 0.961 | 5.57 | 0.71 | 10.84 | **0.030** | 3.79 | -2.85 | 10.65 | 0.246 |
| NRDLS Production SS |  |  |  |  |  |  |  |  |  |  |  |  |
| Arm group | 3.15 | -0.95 | 7.27 | 0.134 | 3.57 | -0.34 | 7.61 | 0.079 | 6.24 | 1.71 | 10.91 | **0.016** |
| Adjusted for Wave |  |  |  |  |  |  |  |  |  |  |  |  |
| Arm group | 3.26 | -1.22 | 7.49 | 0.155 | 4.03 | 0.75 | 7.97 | **0.027** | 6.12 | 1.75 | 10.49 | **0.010** |
| Wave | 1.82 | -2.29 | 5.90 | 0.396 | 5.80 | 2.12 | 9.94 | **0.005** | 6.98 | 2.72 | 11.11 | **0.003** |
| Adjusted for Delayed Intervention |  |  |  |  |  |  |  |  |  |  |  |  |
| Arm group | 3.17 | -1.33 | 7.57 | 0.170 | 3.54 | -0.30 | 7.16 | 0.073 | 6.04 | 1.33 | 10.47 | **0.014** |
| Delayed intervention | -0.86 | -7.48 | 6.01 | 0.794 | 4.73 | 0.47 | 8.46 | 0.022 | 4.59 | -2.39 | 11.97 | 0.218 |

*Key: NRDLS: New Reynell Developmental Language Scales; SS: Standard Score;*
