# Supplementary material for: A Cluster Randomized Controlled Trial Comparing the Efficacy of Pre‐School Language Interventions—Building Early Sentences Therapy and an Adapted Derbyshire Language Scheme
Source: Int J Lang Commun Disord. 2025 Apr 26;60(3):e70036. doi: 10.1111/1460-6984.70036 (PMC12032828; doi:10.1111/1460-6984.70036)
Supplement: Supplementary file 5 — Appendix 5 [file JLCD-60-0-s003.docx]

Appendix 5. Effect Size interpretation guidance recommended by the Education Endowment Foundation (Coe et al 2013)

|  | Effect size | | Description |
| --- | --- | --- | --- |
| Months progress | from | to |  |
| 0 | -0.01 | 11 | Very low or no effect |
| 1 | 0.02 | 0.09 | Low |
| 2 | 0.01 | 0.18 | Low |
| 3 | 0.19 | 0.26 | Moderate |
| 4 | 0.27 | 0.35 | Moderate |
| 5 | 0.36 | 0.44 | Moderate |
| 6 | 0.45 | 0.52 | High |
| 7 | 0.53 | 0.61 | High |
| 8 | 0.62 | 0.69 | High |
| 9 | 0.7 | 0.78 | Very high |
| 10 | 0.79 | 0.87 | Very high |
| 11 | 0.88 | 0.95 | Very high |
| 12 | 0.96 | >1.0 | Very high |
